# Benzisothiazolinone: Pharmacokinetics, Tissue Distribution, and Mass Balance Studies in Rats

**DOI:** 10.3390/metabo13050584

**Published:** 2023-04-23

**Authors:** Seong Jun Jo, Soo Hyeon Bae, Zhouchi Huang, Sangyoung Lee, Chae Bin Lee, Soon Uk Chae, Jung Bae Park, Mihye Kwon, Hye Kyung Chung, Soo Kyung Bae

**Affiliations:** 1 College of Pharmacy and Integrated Research Institute of Pharmaceutical Sciences, The Catholic University of Korea, 43 Jibong-ro, Bucheon-si 14662, Gyeonggi-do, Republic of Korea; seongjun6734@catholic.ac.kr (S.J.J.); zhouchi.huang@pharmacodia.com (Z.H.); nssy0416@catholic.ac.kr (S.L.); aribri727@catholic.ac.kr (C.B.L.); zldtnseo@catholic.ac.kr (S.U.C.); 2 Korea Institute of Radiological & Medical Sciences, Nowon-ro 75, Nowon-gu, Seoul 01812, Republic of Korea; sh.bae@qfitter.com (S.H.B.); sacramentou@naver.com (J.B.P.); kwonmh@kirams.re.kr (M.K.); hkchung@kirams.re.kr (H.K.C.)

**Keywords:** benzisothiazolinone, various routes of exposure, pharmacokinetics, tissue distribution, mass balance

## Abstract

Humans are continuously exposed to benzisothiazolinone (BIT), which is used as a preservative, through multiple routes. BIT is known to be a sensitizer; in particular, dermal contact or aerosol inhalation could affect the local toxicity. In this study, we evaluated the pharmacokinetic properties of BIT in rats following various routes of administration. BIT levels were determined in rat plasma and tissues after oral inhalation and dermal application. Although the digestive system rapidly and completely absorbed orally administered BIT, it underwent severe first-pass effects that prevented high exposure. In an oral dose escalation study (5–50 mg/kg), nonlinear pharmacokinetic properties showed that *C*_max_ and the area under the curve (AUC) increased more than dose proportionality. In the inhalation study, the lungs of rats exposed to BIT aerosols had higher BIT concentrations than the plasma. Additionally, the pharmacokinetic profile of BIT after the dermal application was different; continuous skin absorption without the first-pass effect led to a 2.13-fold increase in bioavailability compared with oral exposure to BIT. The [^14^C]-BIT mass balance study revealed that BIT was extensively metabolized and excreted in the urine. These results can be used in risk assessments to investigate the relationship between BIT exposure and hazardous potential.

## 1. Introduction

An environmental health crisis related to the misuse of biocides (methylisothiazolinone/chloromethlyisothiazolinone (MIT/CMIT) mixture) in humidifier disinfectants has occurred in Korea. It caused 78 casualties, including 36 infants, because of fatal lung disease [1,2]. Both compounds were listed as toxic substances by the Korea Chemical Management Association (KCMA) in November 2012. However, human exposure to isothiazolinones, including CMIT/MIT, is still possible through consumer product use [1,3].

Benzisothiazolinone (1,2-Benzisothiazol-3(2H)-one, BIT), an isothiazolinone-based biocide similar to CMIT and MIT, is widely used as an antimicrobial agent and chemical preservative in water-based solutions, including pastes, paints, and cutting oils [4,5]. Because BIT is present in various products, such as air fresheners, cleaners, stain removers, laundry and dishwashing detergents, hand wash products, and ink cartridges, it can be absorbed through various exposure routes, including oral, dermal, and inhalation [6,7].

BIT is a contact allergen, and its most common side effects are irritation and sensitization. It is a severe eye irritant in rabbits and a moderate contact sensitizer in guinea pigs [5]. The inhalation of BIT by mice induces MUC5AC expression, which is involved in mucus hypersecretion in airway inflammatory diseases [8]. BIT also causes allergic contact dermatitis, which can occur even with very low allergen exposure once an individual is sensitized [4,6].

Although BIT causes contact irritation and sensitization, the regulation of its use in cosmetics remains controversial. According to the ‘Opinion on benzisothiazolinone’ published by the Scientific Committee on Consumer Safety (SCCS), the use of BIT in cosmetics is considered unsafe with respect to sensitization since its safe levels of exposure have not been established [5]. Therefore, BIT has been banned from cosmetics in the EU and Switzerland. However, BIT is being used in cosmetics in Australia, the US, and Canada [7,9].

In toxicology studies, the no observed adverse effect levels (NOAELs) of BIT after oral administration of a specific BIT product containing 84.2% BIT to rats were reported as 12.63 mg/kg/day in a 28-day repeated dose study and 8.42 mg/kg/day in a 90-day repeated dose study [5]. These were based on histopathological lesions observed in the non-glandular stomach, most likely due to BIT being an irritant [5]. However, when considering products containing BIT, the potential for dermal and inhalation exposure to BIT is higher than oral exposure. Nevertheless, pharmacokinetic and toxicokinetic characteristics related to dermal and inhalation exposure have rarely been reported, and quantitative safety levels have not been determined. For an accurate risk assessment of BIT, the relationship between exposure (i.e., concentration) and toxicity should be established. Physiologically based pharmacokinetic modeling is a useful tool for simulating the pharmacokinetic profiles of BIT in plasma as well as target organs.

In this study, we investigated the pharmacokinetic characteristics of BIT as the first step in determining safe BIT levels in humans after inhalation and dermal exposure. Specifically, pharmacokinetic and tissue distribution studies after BIT exposure through various routes, such as oral, dermal, and inhalation, were conducted using rats. A mass balance study of BIT using [^14^C]-BIT was also conducted to investigate the distribution and excretion of BIT.

## 2. Materials and Methods

### 2.1. Materials and Reagents

**Figure 1:**
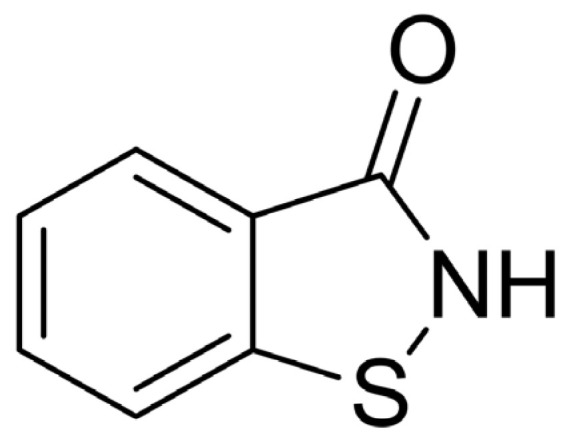
Chemical structure of benzisothiazolinone (BIT).

BIT (purity 97%, Figure 1), dimethyl sulfoxide, formic acid, lanolin, petrolatum, stearic acid, propylparaben, methylparaben, disodium edentate, propylene glycol, triethanolamine, and phenacetin (internal standard, IS, purity ≥ 98%) were purchased from Sigma-Aldrich (St. Louis, MO, USA). The American Radiolabeled Chemicals, Inc. (St. Louis, MO, USA) provided the BIT used for the synthesis of [^14^C]-BIT (specific activity: 55 mCi/mmol;). Ultima Gold and [^14^C] quenching standards were purchased from PerkinElmer (Waltham, MA, USA). Homosalate was purchased from Tokyo Chemical Industry (Tokyo, Japan). High-performance liquid chromatography-grade methanol, ethyl alcohol, and ethyl acetate were obtained from Burdick & Jackson Co. (Muskegon, MI, USA). Deionized water was prepared using a Milli-Q Plus Ultrapure Water System (MilliporeSigma, Burlington, MA, USA).

### 2.2. Animals

Male and female Sprague–Dawley rats (8–10 weeks old, weighing 250–310 g) were purchased from Orient Bio (Seongnam, Gyeonggi-do, Republic of Korea). The protocol for this animal study was approved by the Department of Laboratory Animals, Institutional Animal Care and Use Committee, on the Songsim Campus of the Catholic University of Korea (Approval No. 2019-022) and the Korea Institute of Radiological & Medical Sciences (Approval No. Kirams 2019-0045 for the mass balance study). The rats were maintained in a controlled environment (temperature, 20 ± 2 °C; relative humidity, 55 ± 5%; 12-h light/dark cycle). The animals were used for pharmacokinetic and mass balance studies after an acclimatization period of 1 week with free access to water and feed.

### 2.3. Pharmacokinetic Studies of BIT in Rats

#### 2.3.1. Oral Administration of BIT in Rats

The carotid arteries of each rat were cannulated with polyethylene tubing 50 for blood sampling after a single oral administration. BIT (dissolved in dimethyl sulfoxide: ethanol: distilled water = 10:15:75, *v*:*v*:*v*) at doses of 5, 10, and 50 mg/kg was administered orally to rats (*n* = 6, each) using a gastric gavage tube after overnight fasting with free access to water. The detailed procedures used for anesthesia and handling were previously reported [10]. Blood samples were collected at 0, 3, 5, 15, 30, 45, 60, 90, 120, 180, 240, 360, 480, and 600 min after administration. A heparinized 0.9% NaCl-injectable solution (20 units/mL, 0.3 mL) was used for flushing the cannula immediately after each sampling event to prevent blood clotting. The blood samples were immediately centrifuged at 18,000× *g* for 10 min at 4 °C, and the plasma samples (50 μL) were stored at −80 °C until LC–MS/MS analysis. Biological urine and fecal samples were collected 24 h after BIT administration. Each metabolic cage was rinsed with 10 mL of distilled water, and the rinse water was combined with a 24-h urine sample to determine the exact volume of each sample. Two aliquots of 50 μL of the sample were then stored at −80 °C until LC–MS/MS analysis. Each rat was euthanized with CO_2_ to collect fecal samples. The entire gastrointestinal tract, including its contents and feces, was removed, transferred to a beaker containing 50 mL methanol, and cut into small pieces using scissors. After sonication for 20 min, duplicate aliquots of 50 μL of the supernatant were collected from each beaker and stored at −80 °C until use in the LC–MS/MS analysis.

For multiple oral doses, rats were randomly divided into two groups (*n* = 8 per group) to evaluate the effect of the solvent (dimethyl sulfoxide, ethanol, and distilled water = 10:15:75, *v*:*v*:*v*) on the pharmacokinetics of BIT. Specifically, one group was administered BIT at a dose of 10 mg/kg once daily for 7 days, and the other group (referred to as the ‘vehicle group’) was administered the solvent once a day for 6 days and 10 mg/kg BIT on the 7th day. Blood samples were collected at 0, 3, 5, 15, 30, 45, 60, 90, 120, 180, 240, 360, 480, and 600 min after the last dosing of BIT. Plasma sample preparation procedures were the same as those used for the single oral administration studies.

#### 2.3.2. Dermal Application

For dermal applications, the lotion containing the BIT was freshly prepared 24 h before the pharmacokinetic study, as previously described [11]. The dermal application study was conducted using female rats, according to the Organization for Economic Cooperation and Development (OECD) guidelines for in vivo skin absorption tests (OECD, 2004). A patch containing 30 mg of BIT lotion was dermally applied to the rats (*n* = 8) for 4 h. Blood samples (0.12 mL) were collected from the carotid artery at 0, 90, and 240 min before removing the patches and at 3, 5, 15, 30, 45, 60, 120, 180, 240, and 1200 min after patch removal. In addition, used patches were collected to analyze the amount of BIT remaining in the patches. The blood samples were immediately centrifuged at 18,000× *g* for 5 min, and the plasma samples (50 μL) were stored at −80 °C until LC–MS/MS analysis. The other procedures used for the dermal application study were similar to those in [11].

### 2.4. Inhalation Exposure Study

#### 2.4.1. Inhalation-Delivered Dose Calculation

The inhalation dose concentration (targeted) was calculated using the average body weight of the test animals using the following formula [12]:

$$\mathrm{DD}=\frac{C\;\times \mathrm{RMV}\;\times D}{\mathrm{BW}}$$

where DD = delivered dose (mg/kg); C = concentration of the substance in the air (mg/L); RMV = respiratory minute volume (the volume of air inhaled in one minute; L/min); D = duration of exposure to the aerosol (min); and BW = average bodyweight of rats (0.3 kg). The RMV for rats was calculated using the following formula [12]:

$$\mathrm{RMV}=0.608\times \mathrm{BW}\;{\left(\mathrm{kg}\right)}^{0.852}$$

#### 2.4.2. Inhalation Exposure

Each group (*n* = 3) was exposed to the BIT solution aerosols for 30, 60, 120, or 240 min in a nose-only inhalation chamber (Vitals, Daejeon, Republic of Korea). The detailed exposure system was reported previously [13]. The rats were placed in restraining cones with an internal volume of 5.6 L and individually housed in polycarbonate holding tubes sealed in a chamber with an O-ring during exposure. After the respective exposure times, the rats were removed from the chamber, and blood samples (0.5 mL) were collected via the jugular vein using a heparinized syringe. Subsequently, blood samples were collected as described previously, and the rats were euthanized with CO_2_ gas. After completion of systemic perfusion of the rates with a 0.9% injectable NaCl solution, the livers, lungs, and spleens were excised, washed with a 0.9% injectable NaCl solution, blotted dry with tissue paper, weighed, and homogenized in a 0.9% injectable NaCl solution at 1:2 (*w*:*v*) using a tissue homogenizer. Duplicate aliquots (50 μL) of the homogenates were collected and stored at −80 °C until use in the LC–MS/MS analysis.

### 2.5. Disposition Studies of BIT in Rats

#### 2.5.1. Tissue Distribution Studies after Oral Administration of BIT in Rats

BIT was dissolved in the same vehicle used in the pharmacokinetic studies and administered at a dose of 10 mg/kg. Blood was collected from the carotid artery of each rat (*n* = 5) 30 and 180 min after administration, and each rat was euthanized with CO_2_ gas. Blood samples were prepared and collected as described in the ‘Oral Administration of BIT in Rats’ section. The overall procedure for preparing tissue samples for the analysis of BIT concentrations in the brain, heart, kidney, liver, lungs, and spleen was the same as that described in the ‘Inhalation Exposure’ section. The plasma and tissue BIT concentrations were measured, and tissue-to-plasma partition coefficients (k_p_) were calculated for each tissue.

#### 2.5.2. Mass Balance Study of [^14^C]-BIT in Rats

A mass balance study of [^14^C]-BIT was performed to elucidate its excretion pathway after intravenous, oral, and dermal administration. The biliary excretion of BIT has also been investigated in bile duct cannulated (BDC) rats. As a micro tracer, [^14^C]-BIT (40 μci/kg) was used, and its specific activity was measured the day before the experiment. Dosing solutions were prepared by mixing [^14^C]-BIT and unlabeled BIT in the aqueous solution used in the pharmacokinetic studies. BIT was administered intravenously, orally, or dermally to the rats at doses of 1, 10, or 30 mg/kg. For the bile duct-cannulated rat study, the bile duct was cannulated under anesthesia using polyethylene tubing 10 (Clay Adams, Franklin Lakes, NJ, USA). After BIT dosing, urine, feces, or bile were collected at 0–4, 4–8, 8–12, 12–24, 24–48, and 48–72 h; after 72 h, each metabolic cage was swabbed, and the swabs were also kept for analysis. In the dermal application study, patches were removed from each rat, and swabs were used to wipe around the skin after patch removal. These were collected to analyze the unabsorbed amount of [^14^C]-BIT. The collected samples were weighed and stored at −20 °C until use in liquid scintillation counting (LSC) analysis.

An LSC system (Tri-Carb^®^ 5110 TR; PerkinElmer) was used to confirm the specific activity and analyze the radioactivity of [^14^C]-BIT. Samples were prepared according to the manufacturer’s instructions (PerkinElmer Wallac, Gaithersburg, MD, USA). Briefly, a 0.1-mL aliquot of the collected sample was mixed with 14.9 mL of a scintillation cocktail (Ultima Gold, PerkinElmer) by vortexing for 5 min. Each sample was analyzed for 5 min. The total radioactivity of [^14^C]-BIT was quantified using a [^14^C] quenching standard.

### 2.6. LC–MS/MS Analytical Method for the Quantification of BIT in Biological Samples

Quantification of BIT in all the biological samples obtained in this study was performed using our previously developed and validated LC–MS/MS analytical method [11]. The lower limits of quantification (LLOQs) in plasma, urine, gastrointestinal tract samples, and tissue homogenates were 2 ng/mL, 2 ng/mL, 10 ng/g, and 10 ng/g, respectively.

### 2.7. Pharmacokinetic and Statistical Analysis

Non-compartmental analysis (WinNonlin^®^, version 5.2; Pharsight Corporation, Mountain View, CA, USA) was conducted to calculate the following pharmacokinetic parameters: the total area under the plasma concentration-time curve from time zero to time infinity (AUC_inf_) or to the last measured time (AUC_t_), total body clearance (CL), terminal half-life (*t*_1/2_), mean residence time (MRT), apparent volume of distribution at steady state (Vd_ss_), peak plasma concentration (*C*_max_), time to reach *C*_max_ (*T*_max_), and absolute bioavailability (*F*) [14].

Statistical significance was determined at *p* < 0.05, using a *t*-test between the two means for unpaired data, Duncan’s multiple range test of the Statistical Package for the Social Sciences (SPSS, version 25; IBM Corp., Armonk, NY, USA), or a posteriori analysis of variance among the three means for unpaired data. All results are expressed as the mean ± standard deviation (SD), except for *T*_max_, which was described as the median (range).

## 3. Results

### 3.1. Pharmacokinetic Studies of BIT in Rats

#### 3.1.1. Oral Administration of BIT in Rats

**Figure 2:**
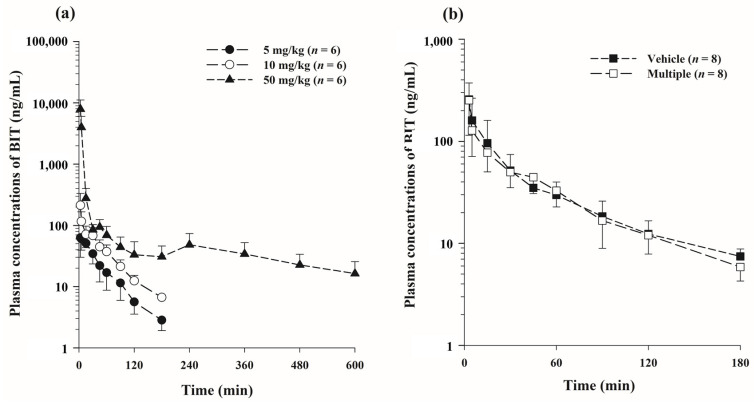
Mean plasma concentration-time profiles of BIT after single oral administration of 5 mg/kg (●), 10 mg/kg (○), and 50 mg/kg (▲) (**a**); multiple oral administration of 10 mg/kg (■) and vehicle group (□) (**b**). Vertical bars represent standard deviations.

The plasma concentration-time profiles of BIT after single (5, 10, and 50 mg/kg) or multiple (10 mg/kg for 7 days) oral doses are shown in Figure 2, and the relevant pharmacokinetic parameters are summarized in Table 1. After single and multiple oral administrations, BIT was rapidly absorbed and detected at high concentrations in the plasma from the first blood sampling time point (3 min), reaching *T*_max_ after 3–30 min. The GI_24 h_ after oral dosing was found to be less than 0.870% of the BIT dose in all dose groups (Table 1). In addition, the urinary excretion of BIT was negligible, with Ae_0–24 h_ values less than 0.0430% after a single administration of BIT. At 50 mg/kg, BIT exhibited non-linear pharmacokinetic properties. The dose-normalized AUC_t_ (AUC_inf_) values of BIT at 50 mg/kg were significantly higher than those at 5 and 10 mg/kg, and similar patterns were observed for the dose-normalized *C*_max_ of BIT (Table 1). A second peak and delayed elimination of BIT were observed in the 50 mg/kg dose group, resulting in a longer half-life. The *t*_1/2_ value of 226 ± 43.6 min at a dose of 50 mg/kg was significantly longer than those of 46.8 ± 7.60 min and 53.9 ± 10.4 min at doses of 5 mg/kg and 10 mg/kg, respectively.

There were no significant differences (*p* > 0.05) in any of the pharmacokinetic parameters of BIT at a dose of 10 mg/kg between the multiple dosing and vehicle groups (Table 1). These results indicate that once-daily administration of BIT does not result in accumulation in the body, that no auto-induction or time-dependent inhibition occurs after multiple doses of BIT, and that additionally, the oral vehicle does not affect the pharmacokinetic profiles of BIT.

#### 3.1.2. Dermal Application of BIT in Rats

**Figure 3:**
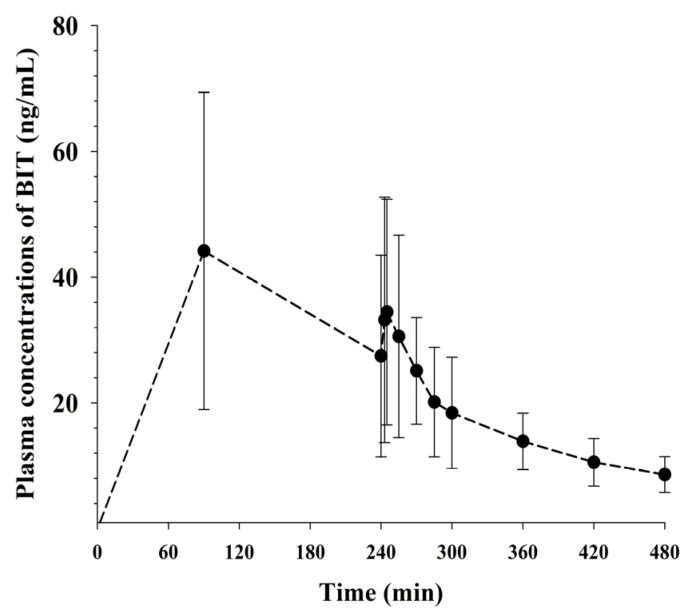
Mean plasma concentration-time profiles of BIT after dermal administration of 30 mg to rats (*n* = 8). Vertical bars represent standard deviations.

The plasma concentration-time profile of BIT following the dermal application of 30 mg BIT is shown in Figure 3, and the corresponding pharmacokinetic parameters are listed in Table 2. Similar to oral administration, BIT reached *T*_max_ rapidly enough to be observed when the patch was attached, which was attributed to the rapid elimination of BIT from the body (Table 2). After 4 h of dermal application, the remaining and/or washed amount found at the application skin site was subtracted from the dermal application amount; this was considered the actual amount absorbed into the systemic circulation. Approximately 9.61% (2.88 ± 0.136 mg/rat) of the dermally applied dose penetrated the dorsal skin.

#### 3.1.3. Inhalation Exposure of BIT in Rats

This study was conducted to investigate the BIT concentrations in the plasma, lungs, liver, and spleen following single inhalation exposure of rats to BIT aerosols. A nose-only inhalation chamber system was used, and an aerosol-containing BIT was generated using a nebulizer. Air was supplied to the nebulizer at 12 L/min, and the nebulizer nebulized the liquid solutions through small apertures by applying compressed dry air at 20–25 mL/h, producing fine aerosols. Because BIT is not soluble in distilled water, a fresh stock solution was prepared at 3 mg/mL using a solvent of the same composition as that used in the oral administration study. The calculated DD was approximately 4 mg/kg/h (3.63–4.54 mg/kg), and the total inhaled doses were determined for each BIT inhalation duration. Thus, if the inhalation durations were 30, 60, 120, and 240 min, the expected BIT inhalation doses were 2, 4, 8, and 16 mg/kg, respectively.

**Figure 4:**
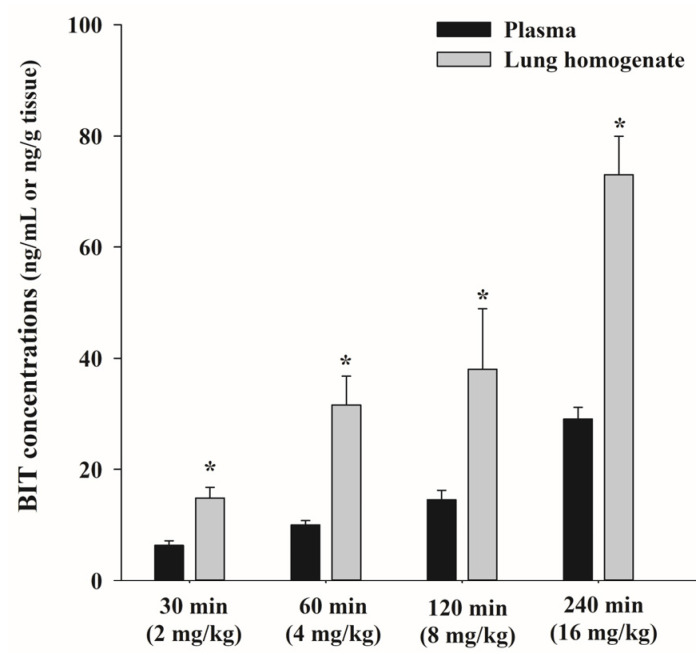
Mean plasma and lung homogenate concentration-time profiles of BIT after inhalation administration to rats (*n* = 3). Vertical bars represent standard deviations. * *p* < 0.05 compared to plasma levels of BIT.

As depicted in Figure 4, the plasma BIT concentrations increased less than proportionally over the inhaled dose ranges, 2–16 mg/kg; the values were 6.30 ± 0.844 ng/mL and 29.1 ± 2.12 ng/mL for 2 and 16 mg/kg, respectively. Similar patterns were observed in the lung tissue homogenates, with values of 14.9 ± 1.92 ng/g tissue and 73.0 ± 6.94 ng/g tissue for 2 and 16 mg/kg, respectively. The lung k_p_ values of BIT after inhalation were 2.36, 3.16, 2.61, and 2.51 at a dose of 2, 4, 8, and 16 mg/kg, respectively.

### 3.2. Disposition Studies of BIT in Rats

#### 3.2.1. Tissue Distribution Studies after Oral Administration of BIT in Rats

**Figure 5:**
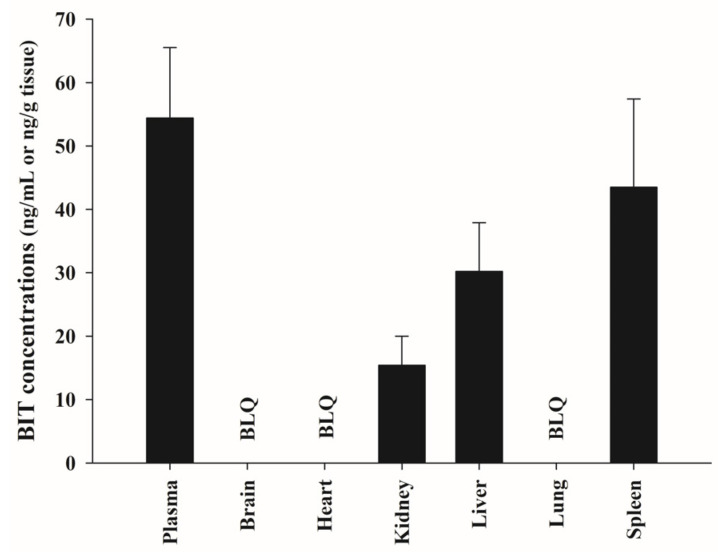
Mean concentration of BIT in plasma and various tissues at 30 min (*n* = 3) after single oral administration of 10 mg/kg. Vertical bars represent standard deviations. BLQ, below the lower limit of quantification.

As shown in Figure 5, 30 min after oral administration of BIT, the highest BIT concentrations were found in the spleen, followed by the liver and kidney homogenates. The levels were 43.5 ± 13.9 ng/g tissue, 30.2 ± 7.71 ng/g tissue, and 15.4 ± 4.61 ng/g tissue, respectively. BIT was detected below the LLOQ (BLQ), or rarely, in the brain, heart, or lung homogenates. As the plasma BIT levels at 30 min were 54.4 ± 11.1 ng/mL, the calculated k_p_ of the spleen, liver, and kidney were also <1.0. However, the BIT concentration in rat tissues 180 min after administration was BLQ.

#### 3.2.2. Mass Balance Study of [^14^C]-BIT in Rats

**Figure 6:**
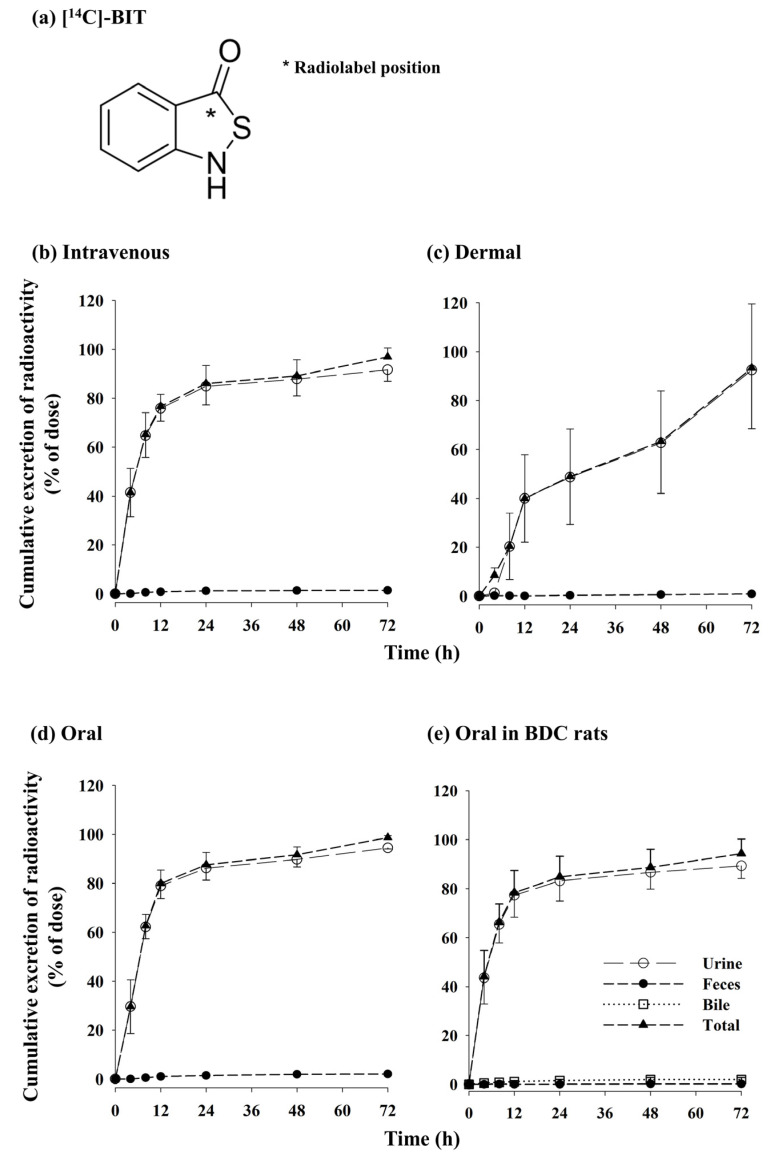
Position of radiolabel on [^14^C]-BIT (**a**). Mean percentage cumulative excretion of radioactivity in urine (○), feces (●), and total (▲) recovery over time for BIT to rats, following intravenous administration of 1 mg/kg BIT (**b**), dermal application of 30 mg BIT (**c**), and oral administration of 10 mg/kg BIT (**d**); mean percentage cumulative excretion of radioactivity in urine (○), feces (●), bile (□), and total (▲) recovery over time for BIT to bile duct cannulated (BDC) rats, following oral administration of 10 mg/kg BIT (**e**) (*n* = 5).

The percentages of cumulative radioactivity excreted in the urine, feces, and bile (only for BDC rats) are shown in Figure 6, and the values are listed in Table 3. The predominant route of excretion of the administered radioactivity (more than 90% of the dose) was via urine in all dosing groups. The total urinary elimination of [^14^C]-BIT-derived radioactivity from the intravenously dosed rats accounted for 91.7 ± 4.76% of the administered dose during the collection period of 0–72 h, with most (82.7 ± 2.38%) of the urinary excretion occurring within the first 12 h after dosing (Figure 6b), indicating a very rapid urinary elimination of BIT. Similar patterns were observed after oral administration of [^14^C]-BIT, with 94.4 ± 1.44% and 83.7 ± 5.58% of the administered radiation recovered in the urine over 72 h and within 12 h post-dose, respectively (Figure 6c). Conversely, fecal excretion was limited, accounting for 1.38 ± 0.806% and 2.05 ± 0.347% of the intravenously and orally administered doses, respectively, by the end of the study (72 h post administration).

After oral administration of [^14^C]-BIT to BDC rats (*n* = 5), 0.146 ± 0.233%, 2.00 ± 0.863%, and 89.3 ± 5.21% of the radioactive dose were excreted in the feces, bile, and urine, respectively, over the 72-h collection period (Figure 6d). A small percentage of BIT and its metabolites were excreted in bile (2.00 ± 0.863%).

When [^14^C]-BIT was dermally applied to rats (*n* = 5), only 7.4% of the dermally applied dose was absorbed systemically, whereas 92.6% of the dose remained in the patch and on the skin, and 0.849 ± 0.393% and 92.5 ± 24.0% of the radioactive dose were excreted in the feces and urine, respectively, over the 72-h collection period (Figure 6e).

Summarizing the results of the mass balance study, most of [^14^C]-BIT and its derivatives were excreted in the urine, indicating that BIT is extensively absorbed systemically after extravascular administrations. However, since exposure to BIT is low and the elimination rate of BIT is fast, it is considered that extensively absorbed BIT is rapidly metabolized and then excreted in the urine as metabolites.

## 4. Discussion

BIT, an isothiazolinone-based biocide, is widely used as a chemical preservative in various products; therefore, it can be absorbed through various routes of exposure, including oral, dermal, and inhalation. In this study, the pharmacokinetic properties of BIT in rats after oral, dermal, and inhalation administration were investigated, and tissue distribution and mass balance studies of BIT were performed to elucidate the disposition of BIT in plasma and tissue.

The oral NOAEL of BIT in rats after 28-day and 90-day exposure was reported to be 15 and 10 mg/kg/day, respectively [5]. Therefore, the pharmacokinetic properties of BIT after a single oral administration at doses of 5, 10, and 50 mg/kg and multiple oral administrations at a dose of 10 mg/kg once daily for seven days in rats were examined in this study. After single or multiple oral administrations, BIT exhibited very rapid and almost complete absorption from the gastrointestinal tract, considering the very fast *T*_max_ range (3–30 min) and negligible GI_24 h_ values (less than 0.870%) in rats (Table 1). In addition, the negligible Ae_0–24 h_ values (less than 0.0430%) suggest that the absorbed BIT undergoes extensive metabolism. Although BIT undergoes rapid and complete absorption in the gastrointestinal tract, it has a significant intestinal and/or hepatic first-pass effect. This effect limits the oral bioavailability (*F*) of BIT (1.78–3.61%, calculated using AUC_inf_ after intravenous dosing, published by Jo et al. [11]), whereas the *F* values vary with oral doses, indicating nonlinear pharmacokinetics. Nonlinear pharmacokinetic profiles of BIT with greater than dose-dependent increases in *C*_max_ and AUC_t_ or AUC_inf_ were observed after an oral dose of 5–50 mg/kg, which may be related to the saturation of the first-pass metabolism of BIT in rats at a higher dose of 50 mg/kg (Table 1). Thus, the nonlinearity in the oral pharmacokinetics of BIT at 50 mg/kg may result from the saturation of the intestinal and/or hepatic first-pass effects. The *t*_1/2_ value of 226 ± 43.6 min at a dose of 50 mg/kg was significantly longer than that at doses of 5 mg/kg and 10 mg/kg (Table 1). Additionally, as shown in Figure 2a, at a high dose of 50 mg/kg, a second peak feature of BIT in rats was found at approximately 240 min, which is typically attributed to enterohepatic circulation and/or multi-site absorption. However, the excretion of BIT and its derivatives via feces and bile was negligible in the mass balance study, and the second-peak feature of the enterohepatic circulation of BIT seems unlikely. Furthermore, we designed the multiple oral administration study at two doses (10 and 50 mg/kg), but BIT administered orally once a day at 50 mg/kg to rats resulted in a survival rate of 12.5% (1/8 rats) within the first three days, thus multiple high oral doses were discontinued (Table S1). In contrast, multiple low oral doses were generally well tolerated and did not result in mortality. The higher incidence of mortality with a high dose is likely associated with the plasma levels, *C*_max_, and AUC because over the higher dose ranges of BIT (50 mg/kg), the plasma levels were dose-proportionally greater than those at 5 mg/kg and 10 mg/kg doses (Table 1). However, the pharmacokinetic parameters of BIT after multiple low doses of 10 mg/kg/day for seven days were similar to those after a single dose of 10 mg/kg in rats, indicating that no time-dependent auto-induction or auto-inhibition occurred after multiple doses of BIT at a low dose (Figure 2; Table 1).

Dermal or inhalation absorption may be more obvious exposure routes for personal care products and household cleaning agents such as BIT than ingestion or intravascular injection. Dermal exposure to BIT as a lotion formulation exhibited a considerably longer *t*_1/2_ value (165 ± 51.9 min), resulting in 2.13-fold increased *F* values compared with oral exposure to BIT (Table 2). This was considered to be due to continuous absorption from the patch applied to the skin. Continuous absorption through the skin prevents extensive first-pass metabolism in the small intestine; thus, the plasma profiles of BIT after dermal application may be similar to those following continuous intravenous infusion [15,16]. However, in the case of toxic chemicals, such as BIT, this characteristic can aggravate exposure to toxicity. From this perspective, the increased *F* after dermal application could be more harmful than the low oral doses of 5 mg/kg and 10 mg/kg because dermal application avoids a significant intestinal first-pass effect, causing prolonged exposure. Confounding factors, such as the exposure time of the dermal application and the application area, may affect BIT concentration in the body. Further pharmacokinetic studies of BIT after the application of higher dermal doses or multiple dermal exposures are required.

In this study, rats exposed to BIT aerosols (range of 2–16 mg/kg) using a nose-only inhalation chamber system exhibited relatively low plasma concentrations (6.36–29.1 ng/mL), which reflect approximately *C*_max_ values at the respective doses. Furthermore, the plasma BIT concentrations increased (approximately 4.57-fold) less proportionally to the inhaled dose range of 2–16 mg/kg. This may be due to the low pulmonary absorption of BIT. Generally, water solubility is one of the key determinants of pulmonary absorption [17], but BIT has a relatively low aqueous solubility (0.8 mg/mL). Conversely, MIT and CMIT, which exhibit high lung toxicity following inhalation administration, have a much higher aqueous solubility (a mixture of MIT/CMIT, 367 mg/mL) than BIT. Another possible reason is the particle size of the inhalation aerosols. Particle size is a major determinant of inhaled drug deposition in the lungs. For efficient lung deposition, the particle size should be 1–5 μm, but we did not check the particle size during the inhalation study. In contrast to its plasma levels, 2.36–3.16-fold higher BIT concentrations were observed in lung tissues. The high distribution into lung tissues might be because BIT is not efficiently absorbed into the systemic circulation, so the unabsorbed proportion remains.

Based on the mass balance study of [^14^C]-BIT after intravenous, dermal, and oral dosing, the total urinary elimination of [^14^C]-BIT-derived radioactivity accounted for 91.7%, 92.2%, and 94.4% of the respective administered dose (absorbed dose for dermal administration) over a 72-h period (Figure 6; Table 3). In contrast, fecal excretion was limited, accounting for 1.38%, 0.849%, and 2.05% of the intravenous, dermal, and orally administered doses, respectively (Figure 6; Table 3). Following oral administration of [^14^C]-BIT to bile duct cannulated rats, values of 0.146%, 2.00%, and 89.3% of the radioactive dose were excreted in the feces, bile, and urine, respectively, over the 72-h collection period (Figure 6e; Table 3). Over 72 h, the overall recovery of radioactivity from the feces and urine, including cage wash, was nearly 100%. These results combined showed that most of the administered BIT was extensively absorbed, metabolized, and excreted as metabolites in the urine, having hardly any BIT left (0.0466%). Only small amounts were eliminated via bile (~2.00%) and feces (~0.146%) as unchanged BIT and metabolites. The negligible Ae_0–24 h_ and GI_24 h_ values for BIT also support these results. Although we did not conduct metabolite profiling of BIT, further characterization of in vivo BIT metabolites is required to provide better insights into the risk assessment of BIT. Gruvberger and Bruze suggested that glutathione could rapidly inactivate isothiazolinones, such as CMIT and MIT, in aqueous solutions [18]. If the metabolic pathways of the two compounds are similar, glutathione conjugation may affect the concentration of BIT.

Meanwhile, intravenously administered BIT exhibited a considerably smaller volume of distribution (Vd_ss_ = 129 mL/kg) [11] than the total body water of rats (668 mL/kg) [19], indicating very limited tissue distribution and that BIT remains mainly in the vascular system. As shown in Figure 5, BIT was not detected in the brain or heart tissues; negligible BIT levels were detected in the spleen, liver, kidney, and lung tissues when compared with those in the plasma. These findings are consistent with the small Vd_ss_. It is generally recognized that high binding to blood cells or plasma proteins may be one of the factors causing a small Vd_ss_ [20,21]. In addition, we found that BIT was extensively bound to rat plasma proteins (>99.4%), with a mean unbound fraction value of 0.584% at concentrations of 5 and 50 μM (Supplementary Materials, Text S1).

## 5. Conclusions

The assessment of the potential health risks posed by using BIT in personal care products and household cleaning agents requires an understanding of its pharmacokinetics after different routes of exposure. Orally administered BIT was rapidly and completely (>99%) absorbed from the gastrointestinal tract; however, it suffered significant intestinal and/or hepatic first-pass effects, which resulted in a very low oral *F* value with a range of 1.78–3.61%. Nonlinear pharmacokinetic profiles of BIT with dose-proportional increases in *C*_max_ and AUC were observed after oral dosing (5–50 mg/kg), which may be related to the saturation of the first-pass metabolism of BIT in rats at a higher dose (50 mg/kg). However, repeated oral exposure for one week to a low dose of BIT (10 mg/kg) did not cause BIT accumulation in the body. Rats exposed to BIT aerosols (2–16 mg/kg) inhaled using a nose-only inhalation chamber system exhibited relatively low plasma concentrations (6.36–29.1 ng/mL). In contrast to the plasma levels, 2.4–3.2-fold higher BIT concentrations were observed in the lung tissues. Dermal exposure showed a quite different pharmacokinetic profile of BIT, with delayed absorption through the skin and a considerably longer *t*_1/2_, resulting in a 2.13-fold increase in *F* values compared to oral exposure to BIT, which is considered to be due to the continuous absorption of BIT from the patch attached to the skin. Based on a mass balance study of [^14^C]-BIT after intravenous, dermal, and oral dosing, BIT in the body was extensively metabolized and excreted almost exclusively as metabolites in the urine, with smaller amounts eliminated in the bile and/or feces. To our knowledge, this is the first study to report the pharmacokinetics, tissue distribution, and excretion patterns of BIT in rats. Further studies are required to investigate the pharmacokinetic properties of other isothiazolinone biocides and reveal the mechanism of toxicity related to functional respiratory or lung failure. The findings of this study will be useful for investigating the relationship between BIT exposure and its toxic potential in risk assessment studies.

## Figures and Tables

**Table 1:** Pharmacokinetic parameters (mean ± SD) of BIT after single or multiple oral administrations of BIT to rats.

Parameters (Units)	Single			Multiple (10 mg/kg)	
	5 mg/kg (*n* = 6)	10 mg/kg (*n* = 6)	50 mg/kg (*n* = 6)	Vehicle Group (*n* = 8)	For 7 Days (*n* = 8)
AUC_t_ (μg/min/mL)	2.98 ± 0.779	5.97 ± 0.774	59.3 ± 19.4 *	6.03 ± 2.16	5.81 ± 1.24
AUC_inf_ (μg/min/mL)	3.20 ± 0.840	6.44 ± 0.784	64.9 ± 20.9 *	6.59 ± 2.10	6.23 ± 1.29
*t*_1/2_ (min)	46.8 ± 7.60	53.9 ± 10.4	226 ± 43.6 *	52.3 ± 9.91	51.5 ± 13.7
*C*_max_ (ng/mL)	76.1 ± 24.5	219 ± 103	7920 ± 3240 *	257 ± 106	262 ± 125
*T*_max_ (min) ^a^	4 (3–30)	3 (3–30)	3 (3–5)	3 (3–5)	3 (3–15)
GI_24 h_ (% of dose) ^b^	0.0874 ± 0.0483	0.364 ± 0.251	0.870 ± 0.491	0.309 ± 0.184	0.269 ± 0.0903
Ae_0–24 h_ (% of dose) ^c^	0.0288 ± 0.00721	0.0397 ± 0.00813	0.0430 ± 0.0108	0.0363 ± 0.0726	0.0510 ± 0.0662
*F* (%) ^d^	1.78	1.79	3.61	1.83	1.73

The dose-normalized AUC and *C*_max_ values based on 5 mg/kg BIT were compared in the statistical analysis. * The 50 mg/kg group was significantly different (*p* < 0.05) from the 5 and 10 mg/kg dosing groups. ^a^ T_max_ is shown as the median (range). ^b^ Percentage of unchanged BIT recovered from the gastrointestinal tract, including its contents and feces, at 24 h. ^c^ Percentage of BIT excreted as an unchanged form over the 24-h urine. ^d^ The values of *F* (%) in each dose were calculated using the AUC_inf_ of BIT after intravenous administration at a dose of 10 mg/kg, the results of which were published by Jo et al. [11].

**Table 2:** Pharmacokinetic parameters (mean ± SD) of BIT after dermal application of BIT to rats.

Parameters (Units)	Dermal Application Amount
	30 mg/rat (*n* = 8)
Dermal absorbed amount for 4 h (mg)	2.88 ± 0.136
AUC_t_ (μg/min/mL)	11.4 ± 4.45
AUC_inf_ (μg/min/mL)	14.0 ± 5.55
*t*_1/2_ (min)	165 ± 51.9
*C*_max_ (ng/mL)	47.0 ± 23.2
*T*_max_ (min) ^a^	90 (90–255)
Relative *F* (%) ^b^	213

The amount of dermally applied BIT was 30 mg for each rat; the application area was 7 × 10 cm^2^, covered using a dressing bandage, and the application period was 4 h. ^a^ *T*_max_ is shown as the median (range). ^b^ The relative *F* values after the dermal application of BIT were calculated and compared with the values of AUC_inf_ after oral administration of BIT (10 mg/kg).

**Table 3:** Excretion of total radioactivity after intravenous (1 mg/kg), dermal (30 mg/kg), and oral (10 mg/kg) administration of BIT in intact or bile duct-cannulated rats.

Time Interval (h)	Radioactivity of Excretion (% of Dose)								
	Intravenous		Dermal ^a^		Oral in Intact Rats		Oral in BDC Rats		
	Urinary Excretion	Fecal Excretion	Urinary Excretion	Fecal Excretion	Urinary Excretion	Fecal Excretion	Urinary Excretion	Fecal Excretion	Biliary Excretion
0–4	41.4	0.0139	1.24	0.164	29.6	0	43.6	0	0.490
4–8	64.7	0.528	20.2	0.121	62.2	0.509	65.5	0.0889	0.791
8–12	75.9	0.775	40.0	0.0170	79.0	0.988	77.3	0	1.09
12–24	84.9	1.16	48.7	0.282	86.2	1.43	83.2	0.0231	1.53
48–48	87.9	1.28	62.7	0.593	89.8	1.87	86.7	0.149	1.93
48–72	91.7	1.38	92.5	0.849	94.4	2.05	89.3	0.146	2.00
Swabs ^b^	3.88		7.15		2.23		2.79		

^a^ In dermal studies, the cumulative excretion of radioactivity was calculated using the actual absorbed amount. ^b^ Swabs were collected after cage washing and 72 h after dermal application.

## Data Availability

Not applicable.

## Supplementary Materials

The following supporting information can be downloaded at: https://www.mdpi.com/article/10.3390/metabo13050584/s1, Text S1: Determination of the unbound fraction of BIT in rat plasma [22]; Table S1: Survival rate in rats after oral administration of BIT at 50 mg/kg/day for 7 days.

## Abbreviations

AUC_inf_	Area under the plasma concentration-time curve from time zero to infinity
AUC_t_	Area under the plasma concentration-time curve within the time span of zero to last
Ae_0–24 h_	Percentage of BIT excreted as an unchanged form over the 24-h urine
BDC	Bile duct cannulated
BIT	Benzisothiazolinone
BLQ	Below the lower limit of quantification
*C*_max_	Peak plasma concentration
CMIT	Chloromethylisothiazolinone
*F*	Bioavailability
k_p_	Tissue-to-plasma partition coefficient
LC–MS/MS	Liquid chromatography with tandem mass spectrometry
LLOQ	Lower limit of quantification
LSC	Liquid scintillation counting
MIT	Methylisothiazolinone
NOAEL	No observed adverse effect level
RMV	Represents respiratory minute volume, which is the respiratory rate multiplied by the tidal volume
t_1/2_	Terminal half-life
*T*_max_	Time to reach a *C*_max_
Vd_ss_	Apparent volume of distribution at steady state
