# Supplementary material for: Benzisothiazolinone: Pharmacokinetics, Tissue Distribution, and Mass Balance Studies in Rats
Source: Metabolites. 2023 Apr 23;13(5):584. doi: 10.3390/metabo13050584 (PMC10223580; doi:10.3390/metabo13050584)
Supplement: Supplementary file 1 [file metabolites-13-00584-s001.zip › Table S1.pdf]

Supplementary Table

Benzisothiazolinone: Pharmacokinetics, Tissue Distribution, and Mass Balance studies in Rats

Seong Jun Jo [1,†], Soo Hyeon Bae [2,†,‡], Zhouchi Huang [1], Sangyoung Lee [1], Chae Bin Lee [1], Soon Uk Chae [1], Jung Bae Park [2], Mihye Kwon [2], Hye Kyung Chung [2] and Soo Kyung Bae [1,*]

Table S1. Survival rate in rat after oral administration of BIT at 50 mg/kg/day for 7 days

Day (Number of dose)	Survivors (*n*)	Survival rate (%)
0 (1th)	8/8	100
1 (2nd)	7/8	87.5
2 (3rd)	4/8	50
3 (4 th)	1/8	12.5
