# Supplementary material for: Benzisothiazolinone: Pharmacokinetics, Tissue Distribution, and Mass Balance Studies in Rats
Source: Metabolites. 2023 Apr 23;13(5):584. doi: 10.3390/metabo13050584 (PMC10223580; doi:10.3390/metabo13050584)
Supplement: Supplementary file 1 [file metabolites-13-00584-s001.zip › Text S1.pdf]

# Supplementary Material, Text S1

## Benzisothiazolinone: Pharmacokinetics, Tissue Distribution, and Mass Balance studies in Rats

**Seong Jun Jo [1,†], Soo Hyeon Bae [2,†,‡], Zhouchi Huang [1], Sangyoung Lee [1], Chae Bin Lee [1], Soon Uk Chae [1], Jung Bae Park [2], Mihye Kwon [2], Hye Kyung Chung [2] and Soo Kyung Bae [1,*]**

### Determination of the unbound fraction of BIT in rat plasma

#### Materials & Method

The plasma protein bindings were performed using a rapid equilibrium dialysis device and cellulose membranes with a molecular weight cutoff of 8000 (Thermo Scientific, Rockford, IL, USA) [1]. The rat plasma samples containing BIT at 5 and 50 μM, respectively, were dialyzed against a dialysis buffer, phosphate-buffered saline (PBS, 400 μL). The loaded dialysis plate was covered with sealing tape, placed on an orbital shaker at approximately 200 rpm, and incubated at 37 °C for 4 h. Thereafter, samples (100 μL) from both PBS and plasma chambers were collected and mixed with an equal volume of blank plasma and PBS, respectively. All samples were stored at −80 °C until LC-MS/MS analysis. The unbound fraction of BIT in rat plasma was calculated by dividing the BIT concentration in PBS by that in plasma.

#### Result

BIT was extensively bound to rat plasma proteins. The free fractions (%) of BIT at 5 and 50 μM in rat plasma were 0.562 ± 0.0152% and 0.606 ± 0.0416%, respectively, ($n$ = 3, respectively).

[1] Cho D.Y., Bae S.H., Lee J.K., Kim Y.W., Kim B.T., Bae S.K. Selective inhibition of cytochrome P450 2D6 by Sarpogrelate and its active metabolite, M-1, in human liver microsomes. Drug Metab. Dispos. 2014;42:33–39. doi: 10.1124/dmd.113.054296.
